# ΦX174 Attenuation by Whole-Genome Codon Deoptimization

**DOI:** 10.1093/gbe/evaa214

**Published:** 2020-10-12

**Authors:** James T Van Leuven, Martina M Ederer, Katelyn Burleigh, LuAnn Scott, Randall A Hughes, Vlad Codrea, Andrew D Ellington, Holly A Wichman, Craig R Miller

**Affiliations:** 1 Department of Biological Science, University of Idaho; 2 Institute for Modeling Collaboration and Innovation, University of Idaho; 3 Applied Research Laboratories, University of Texas, Austin; 4 Institute for Cellular and Molecular Biology, University of Texas, Austin; 5 Present address: Seattle Children’s Research Institute, Seattle, WA; 6 Present address: Biotechnology Branch, CCDC US Army Research Laboratory, Adelphi, MD

**Keywords:** bacteriophage, epistasis, fitness landscape, synthetic biology, live-attenuated vaccine, codon bias

## Abstract

Natural selection acting on synonymous mutations in protein-coding genes influences genome composition and evolution. In viruses, introducing synonymous mutations in genes encoding structural proteins can drastically reduce viral growth, providing a means to generate potent, live-attenuated vaccine candidates. However, an improved understanding of what compositional features are under selection and how combinations of synonymous mutations affect viral growth is needed to predictably attenuate viruses and make them resistant to reversion. We systematically recoded all nonoverlapping genes of the bacteriophage ΦX174 with codons rarely used in its *Escherichia coli* host. The fitness of recombinant viruses decreases as additional deoptimizing mutations are made to the genome, although not always linearly, and not consistently across genes. Combining deoptimizing mutations may reduce viral fitness more or less than expected from the effect size of the constituent mutations and we point out difficulties in untangling correlated compositional features. We test our model by optimizing the same genes and find that the relationship between codon usage and fitness does not hold for optimization, suggesting that wild-type ΦX174 is at a fitness optimum. This work highlights the need to better understand how selection acts on patterns of synonymous codon usage across the genome and provides a convenient system to investigate the genetic determinants of virulence.

**Significance**

Attenuating viruses by inserting many synonymous, deleterious mutations offers a means to make potent and reversion-resistant vaccines. We investigate where in a viral genome attenuating mutations should be made and how they should be combined by generating a combinatorial network of codon deoptimized bacteriophage strains. By analyzing the effects of genome editing using mathematical models of epistasis, we find that fitness effects differ between genes in how these deleterious mutations combine. These results show how synonymous mutations can have large effects and will help researchers design synonymously recoded, live-attenuated vaccines.

## Introduction

### Synonymous Compositional Features of Viral Genomes

The unequal use of synonymous codons is known as codon usage bias. Codon biases are the result of an interaction between mutational and selective pressures (Bulmer 1991; Long et al. 2018) and are observed in organisms across the tree of life (Grantham et al. 1980; Ikemura 1985; Hershberg and Petrov 2008). A commonly accepted explanation for why organisms do not evenly use synonymous codons is that the rate of translation (i.e., the amount of proteins being made) is affected by the abundance of tRNAs that pair with codons on a strand of mRNA (Ikemura 1985). Codons that result in the optimal amount of protein then confer a selective advantage. However, there are many other compositional features within the DNA sequences of protein-coding genes upon which selection acts and understanding what features are most influential on selection is a complicated endeavor. These features include: the genic GC content (Raghavan et al. 2012; Kelkar et al. 2015; Newman et al. 2016), CpG or TpA dinucleotides (Burns et al. 2009; Atkinson et al. 2014; Gaunt et al. 2016; Fros et al. 2017; Giallonardo et al. 2017), codon pairs (Gutman and Hatfield 1989; Irwin et al. 1995; Tats et al. 2008; Gamble et al. 2016), endonuclease recognition sites (Karlin et al. 1992; Levin 1993; Rocha et al. 2001; Pleška and Guet 2017), intron splicing motifs (Cáceres and Hurst 2013), mRNA folding stability (Kudla et al. 2009; Presnyak et al. 2015; Boël et al. 2016; Kelsic et al. 2016; Burkhardt et al. 2017; Jack et al. 2019), ribosomal pausing sites (Ponnala 2010; Li et al. 2012), concentration of unpreferred codons at the 5′ ends of transcripts (Chen and Inouye 1990; Tuller et al. 2010; Goodman et al. 2013), autocorrelation of codons on transcripts (Cannarozzi et al. 2010), and capacity of codon order to influence cotranslational folding of proteins (Zhang et al. 2009; Yu et al. 2015). Natural selection acting on one or more of these features can favor the use of certain codons over other synonymous ones. The strength of selection acting on these synonymous codons can be quite strong (Agashe et al. 2016; Lawrie et al. 2013; Bailey et al. 2014; Knöppel et al. 2016; Machado et al. 2017; Kristofich et al. 2018) and some studies compared the relative impact of altering different features (e.g., codon bias vs. mRNA folding). Kudla et al. (2009) generated 154 versions of green fluorescent protein that varied only at synonymous sites and found that mRNA folding around the ribosomal binding site to be most predictive of exogenous green fluorescent protein expression. In an analysis of 6,348 cloned and expressed genes, Boël et al. (2016) found mRNA folding around the translation initiation site to be the second most important predictor of expression level, behind overall codon usage.

As viruses must utilize their hosts’ cellular machinery, there is an expectation that virus genomes are enriched for host-preferred codons to maximize production of viral proteins. This appears to be only partially true. Many viral genomes do contain more host-preferred codons than expected by chance, especially for genes encoding viral structural proteins of dsDNA phages (Carbone 2008; Lucks et al. 2008; Chithambaram et al. 2014). However, many viral genes are not enriched in host-preferred codons. Sometimes unpreferred codons are used to temporally regulate viral gene expression, potentially to avoid host immune responses (Shin et al. 2015). Other virus genomes appear to have little preference for codons abundant in the host genome. For instance, Lucks et al. (2008) found that the majority of 74 bacteriophage genomes show no significant preference for host-preferred codons. Similar discordance between host and viral codon usage patterns are observed in other studies (Kunisawa 1992; Kunisawa et al. 1998; Sau et al. 2005). This discordance could be caused by insufficient selection on codon usage, host–phage relationships that are too short-lived for selection to fine-tune codon usage in the phage, or an inadequate understanding of what features are being selected for. Synonymously editing viral genomes provide an opportunity to learn about viral adaptation to hosts.

### Vaccine Development by Synonymous Recoding

Empirically developed (e.g., serial passage viral adaptation) vaccines have saved millions of lives over the last century, yet methodological improvements make rationally designed, recombinant vaccines attractive because they can be rapidly produced and specifically engineered for safety and effectiveness (Lim et al. 2006; Rueckert and Guzmán 2012; Nabel 2013; Minor 2015; Ramezanpour et al. 2016). One proposed method of generating recombinant vaccines involves making many synonymous, attenuating changes to viral genomes, that is, “deoptimizing” the viral genes (Mueller et al. 2010). The recombinant vaccine can be made by either editing the genome of the wild-type virus or by generating a viral genome entirely from synthesized nucleic acids. Synonymous deoptimization offers a potentially efficient and effective way of making vaccines: the protein sequences of recoded vaccines are identical to their target viruses, they replicate in their host to provide prolonged exposure to the antigen, and the introduction of many synonymous changes presumably assures evolutionary robustness, preventing the evolution of virulence by reversion.

Poliovirus serves as a very good example for the synonymous recoding strategy. Development of a robust, live-attenuated poliovirus vaccine is desired because in some areas on Earth wild poliovirus and the emergent vaccine-derived polioviruses (cVDPVs) continue to cause concern over the resurgence of poliomyelitis (Cann et al. 1984; Kew 2012; Famulare et al. 2016; Jorba et al. 2016). A synthetic poliovirus was assembled in 2002 (Cello et al. 2002), codon deoptimized in 2006 (Burns et al. 2006; Mueller et al. 2006), codon pair deoptimized in 2008 (Coleman et al. 2008), and dinucleotide deoptimized in 2009 (Burns et al. 2009). In all cases, attenuated viruses were produced by recoding the P1/capsid region of the genome. In vitro, these viruses replicate slower and produce lower viral titers than wild-type virus. In vivo, the codon pair deoptimized strain protected mice against challenge by wild poliovirus (Coleman et al. 2008). Although the mechanism of attenuation is not yet fully elucidated, reduced protein expression of the deoptimized genes is observed (Burns et al. 2006, 2009; Mueller et al. 2006; Coleman et al. 2008). These deoptimized poliovirus constructs are genetically stable and remain nonvirulent for up to 25 passages in cell culture (Burns et al. 2006; Coleman et al. 2008).

The apparent success of building poliovirus vaccine candidates using synonymous recoding led to similar attempts to develop vaccines for influenza, adeno-associated, human immunodeficiency, papilloma, chikungunya, respiratory syncytial, simian immunodeficiency, porcine reproductive and respiratory syndrome, echovirus 7, tick-borne encephalitis, vesicular stomatitis, dengue, T7, Lassa, adeno, and swine fever viruses (reviewed in Martínez et al. 2016). The most common method for synonymously deoptimizing viruses is recoding wild-type genes with increased proportions of unpreferred codons (Mueller et al. 2006; Luan et al. 2009; Bull et al. 2012; Cladel et al. 2013; Meng et al. 2014; Nogales et al. 2014; Cheng et al. 2015, 2017; Rostad et al. 2016; Velazquez-Salinas et al. 2016) although other methods of recoding have been successful as well. For example, viral fitness was decreased when synonymous substitutions were randomly introduced (Nougairede et al. 2013; Fabritus et al. 2015, 2016), when codons were replaced by those infrequently used in viral (not host) genes (Burns et al. 2006; Meng et al. 2014), when the proportion of optimal codons was “increased” (Cladel et al. 2013; Vabret et al. 2014; Liang et al. 2017; Villanueva et al. 2016), or when codons were exchanged for codons one substitution away from a translational termination codon (Moratorio et al. 2017).

### No Predictive Understanding of Synonymous Recoding

Although it is clear that synonymous recoding causes attenuation and the strategy holds promise for vaccine development, we lack a predictive understanding of the process. Part of this results from the biological complexity and variation in the systems involved. In many cases, the fitness impact of recoding is cell-line dependent (Martrus et al. 2013; Nougairede et al. 2013; Le Nouën et al. 2014; Meng et al. 2014; Cheng et al. 2015; Shen et al. 2015; Rostad et al. 2016; Velazquez-Salinas et al. 2016), is inconsistent between in vivo and in vitro experiments (Shen et al. 2015; Velazquez-Salinas et al. 2016; Cheng et al. 2017), or is temporally variable (Villanueva et al. 2016). Another obstacle is the nature of the genetic code itself. It is generally challenging to manipulate one synonymous feature of the genome and hold all the others fixed. For example, when codons are shuffled to change codon pair frequency, mRNA stability may be affected, or when codons are deoptimized, codon pair frequencies also change. This makes it difficult to attribute the cause of fitness decreases to one factor (e.g., codon usage adaptation), especially when the features are correlated. As we do here, most studies have focused on manipulating a single compositional feature of the genome and measuring its impact on fitness. Standardizing recoding methodologies and features measured across studies would greatly improve our understanding of the factors that drive fitness decreases and other phenotypic effects caused by synonymous deoptimization.

Despite the optimistic results achieved in studies on synonymous recoding to date, basic questions underlying the method itself remain unanswered (Martínez et al. 2016). What is the best strategy to perform synonymous recoding to achieve attenuation? Can generalities be made about the extent of recoding and the degree of attenuation—or will the biological details and idiosyncrasies of each system preclude this? What is the mechanistic cause of attenuation from synonymous recoding? Are viruses recoded this way robust against fitness recovery? Is it more effective to maximally recode less of the genome (say one gene), or make the recoding less severe, but distribute it across the genome? As an increasing proportion of the genome is recoded, or equivalently, as multiple recoded parts are combined, does attenuation respond in an additive or nonadditive manner? A deeper understanding of genome evolution and synonymous sequence choice is required to answer these questions.

**Fig. 1:**
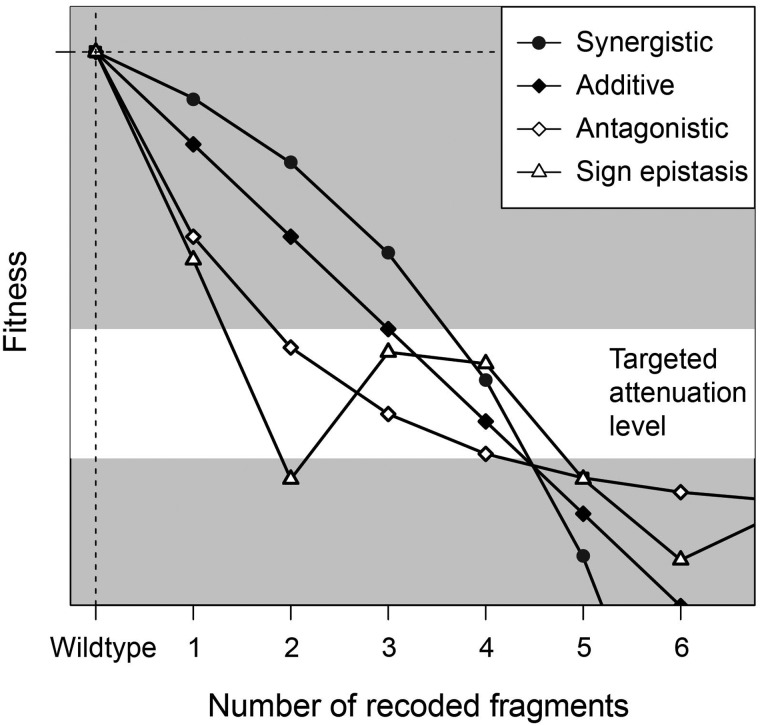
Invented data illustrate that epistasis affects how a desired level of attenuation is achieved. When a substantial amount of attenuation is desired (the “targeted attenuation range” is at a low fitness level), the amount of attenuation (e.g., number of deleterious mutations) will be harder to achieve if mutational effects combine synergistically (negative epistasis) because fitness declines at an increasing rate. In this case, the targeted amount of attenuation will be easier to achieve if mutations combine antagonistically (positive epistasis) because a larger range in the number of deleterious mutations results in the same level of fitness effects. Notice that this pattern is reversed if a slight level of attenuation is desired (near *y* = 0). If sign epistasis, or even irregular magnitude epistasis is observed, then the underlying nature of interactions is more difficult to predict and generalize.

In this article, we focus on two issues related to the codon deoptimization of viruses. First, we seek to compare the fitness effects of mutating different genes in the same virus. Second, we seek to understand how, when attenuating mutations are combined, they interact to affect fitness (i.e., epistasis of deleterious mutations). The nature of epistasis among fragments is crucial for modeling fitness effects: if mutational effects combine synergistically (i.e., the combined fitness being even lower than predicted from the observed individual effects), the range in the number of mutations needed to achieve the targeted attenuation level would tend to be reduced (fig. 1). Conversely, if they combine antagonistically (i.e., the combination of mutations are less attenuated than predicted from individual effects), it may be easier to achieve a target attenuation level, but there may be a limit to how much attenuation is possible. If mutations, in combination, display sign epistasis, irregular magnitude epistasis, or even vary between synergistic and antagonistic epistasis, it will suggest the underlying process is complex and difficult to predict and generalize. To evaluate these issues, herein, we have recoded all the nonoverlapping genes of the bacteriophage **Φ**X174 in fragments, combined recoded fragments in all possible within-gene permutations, and measured the fitness of the resulting recoded bacteriophage.

## Results

### Synonymously Deoptimizing _Φ_X174 Genes

**Fig. 2:**
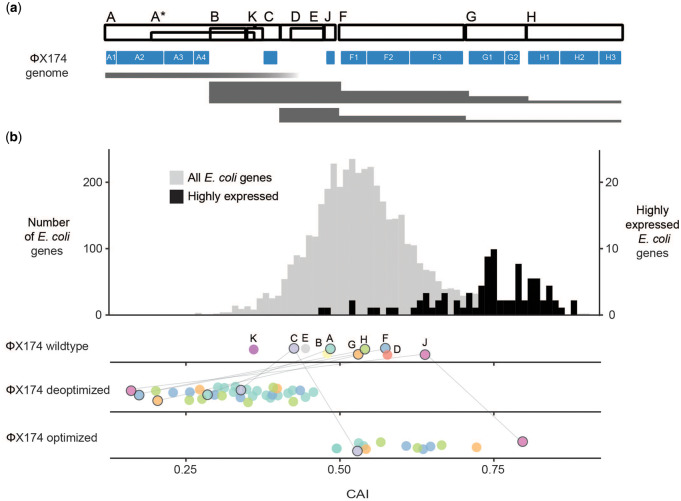
ΦX174 genome organization and capacity for deoptimization relative to host genes. (*a*) Genes on the ΦX174 genome are labeled at the top and shown as white boxes. Recoded regions are shown as filled blue boxes. These fragments are named consecutively (e.g., A1, A2, A3, A4, F1, F2, F3, … ). Transcript expression levels are shown as filled gray bands. The band heights are proportional to the relative number of transcripts by RT–qPCR (Zhao et al. 2012). (*b*) Codon adaptation index (CAI) of *Escherichia coli*, wild-type ΦX174, and recoded ΦX174 genes. Genes highly expressed in *E. coli* are enumerated on the secondary *y* axis. The white space between recoded fragments (the BsmBI sites) was enlarged for visualization. Lines connect genes that were fully recoded. Genes containing only some recoded fragments (e.g., deoptimized A1) do not have black boarders.

**Φ**X174 is a bacteriophage with a 5.4-kb single-stranded DNA genome containing 11 genes (fig. 2*a* and table 1). We measured codon usage bias of **Φ**X174 genes using the codon adaptation index (CAI). CAI is a gene-level statistic running from zero to one that summarizes the extent that codons in a gene are used, rarely (CAI nearer 0) or commonly (CAI nearer 1) among highly expressed host genes (Sharp and Li 1987). We found that most **Φ**X174 genes are not particularly enriched for preferred *Escherichia coli* codons (fig. 2*b*). Only gene J has a CAI value in the upper quartile of *E. coli* genes. Gene K uses the most unpreferred codons and has a CAI value near the lowest value observed for genes in the *E. coli* genome. Genes J and K were the only wild-type **Φ**X174 genes found to be significantly different from simulated genes (Bourret et al. 2019) of similar length using the host’s codon usage preferences (*P* < 0.01, see Supplementary Material online). All other **Φ**X174 genes have CAI values within the range of most *E. coli* protein-coding genes. **Φ**X174 structural proteins (B, D, F, G, H, J) have higher CAI values than nonstructural genes (A, C, E, K), suggesting that high expression of these proteins is important for viral fitness. When we computationally deoptimized entire **Φ**X174 genes (i.e., recoded them to use the least-preferred codons throughout), the resulting CAI values were in the lower tail or even below the tail for all *E. coli* genes (fig. 2 and see Supplementary Material online). These reductions in CAI were the result of changing between 42% (20/48 codons for gene C) and 75% (24/32 codons for gene J) of the codons of a gene, corresponding to 15–32% of its base pairs (supplementary table S1, Supplementary Material online). All the other **Φ**X174 genes fall within this range of recoding (42–75% of codons changed). We calculated additional metrics of codon adaptation including an alternative version of CAI (Xia 2007), tRNA adaptation index: tAI (dos Reis et al. 2004), index of translation elongation: *I*_TE_ (Xia 2015), relative codon adaptation: RCA (Fox and Erill 2010), the number of effective codons: Nc (Wright 1990), COdon Usage Similarity INdex (Bourret et al. 2019), and the starvation codon adaptation index: sCAI (Elf et al. 2003). Nc is a simple index of codon bias that measures the deviation from uniform codon usage. RCA is similar to Nc in that it can be calculated for genes without additional genetic information, but it provides a measure of codon preference that is corrected for gene length and nucleotide content. Like Nc and RCA, tAI does not rely on a list of preferred codons, but it does require the tRNA gene copy number of a genome. A list of highly expressed genes (or the frequency of preferred codons) is needed to calculate both CAI and *I*_TE_, but *I*_TE_ differs from CAI in its handling of R- and Y-ending codon subfamilies. To calculate sCAI, empirical measures of tRNA concentrations are needed. Like CAI and *I*_TE_, COUSIN requires a codon usage table from a user-defined gene set (e.g., highly expressed genes), but is uniquely powerful in comparing codon usage to a random codon null and a reference gene set. Calculation details are in the Materials and Methods section. All analyses produced qualitatively similar rank-orders for **Φ**X174 genes (see Supplementary Material online).

**Table 1:** ΦX174 Gene Function and Protein Copy Number Required for the Assembly of One Virion

Gene	Protein Function	Copies	Gene Length (bp)
A	DNA replication		1,541
A*	DNA packaging, regulation of DNA replication		1,025
**B**	**Internal procapsid scaffolding**	60	362
K	Unknown, not essential		170
C	Regulation of DNA replication		260
D	External procapsid scaffolding	240	458
E	Cell lysis		275
**J**	**DNA packaging**	60	116
**F**	**Major capsid protein**	60	1,283
**G**	**Major spike protein**	60	527
**H**	**Minor spike, pilot protein**	12	986

Note.—Genes encoding structural proteins are bolded.

### Codon Deoptimization of _Φ_X174 Genes Reduces Viral Fitness

**Fig. 3:**
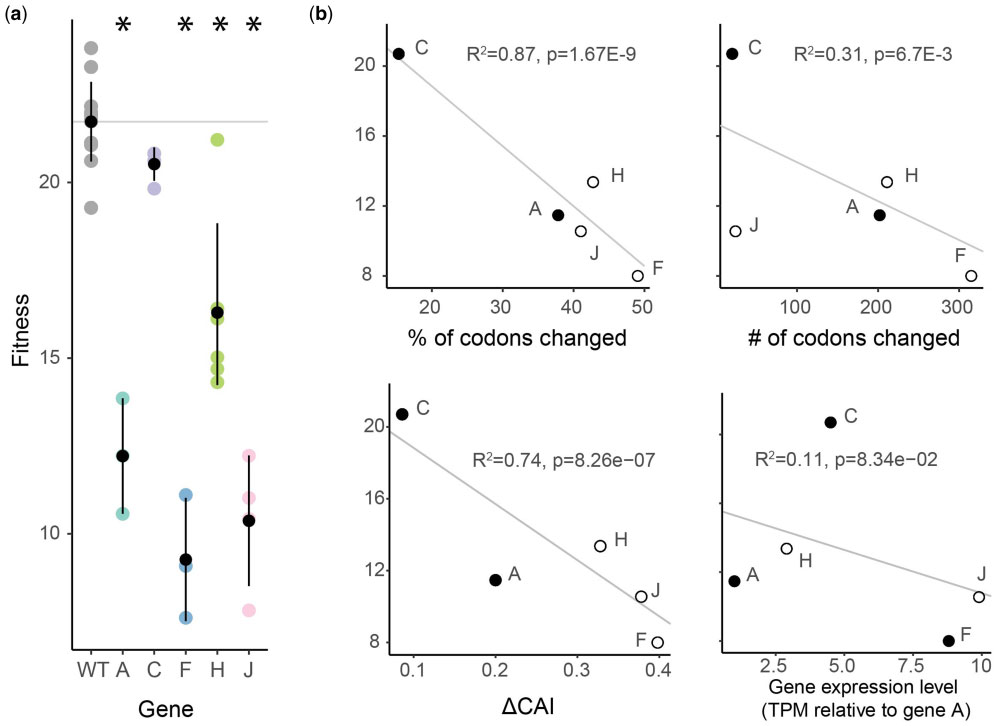
Fitness effects of deoptimizing ΦX174 genes. (*a*) The fitness of wild-type and deoptimized ΦX174 strains containing recoded genes are shown in replicate (colored dots). Means and standard error bars are shown in black. Fitness values that are significantly different from wild type are indicated with asterisks (ANOVA, *P* < 0.01). (*b*) Fitness is plotted against measures that potentially explain fitness decreases. Fitness is the number of doublings per hour (log2 of the ratio of the phage concentration at 60 min divided by the phage concentration at time zero). Gene expression levels are from Logel and Jaschke (2020) and are normalized to gene A. Structural proteins are shown as empty circles. Deoptimizing gene G yielded no viable phage. The total number of independent fitness measurements is provided in Supplementary Material online. At least three replicates were performed for every strain. *y* axis are the same in panels (*a*) and (*b*). The percent of codons changed and change in CAI are highly correlated (*R*^2^ = 0.92).

We codon deoptimized whole **Φ**X174 genes by exchanging wild-type **Φ**X174 codons for synonymous codons less commonly used by its *E. coli* host. Because amino acid usage is not random in **Φ**X174, our recoding resulted in some amino acids being changed synonymously more often than others (supplementary fig. S1, Supplementary Material online). We did not recode regions of genes that overlapped with other genes nor the first six codons of each gene since these codons are known to have strong effects on gene expression (Bentele et al. 2013). This leaves six genes (A, C, J, F, G, H) that could be deoptimized. The construct containing the fully deoptimized G gene could not be recovered, even after growing the strain overnight in an attempt to obtain a recovery mutation. Of the remaining five constructs, four were less fit than wild-type **Φ**X174 (fig. 3*a*). Recoding highly expressed genes (J, F, and G) resulted in larger fitness decreases than recoding lowly expressed genes (A and H). Although the number of variants built was small, the fitness effects of deoptimization were correlated to the amount of recoding performed and the change in CAI of the recoded genes (fig. 3*b* and supplementary table S1, Supplementary Material online). However, the change in CAI is highly correlated with the number of changes made to the gene (*R*^2^ = 0.92) and although a stepwise regression analysis does not suggest removing either from a multivariate model, the variance inflation factor values for the % of codons changed, the number of codons changed, the change in CAI, and the relative expression of wild-type genes are 32.8, 3.8, 31.4, and 3.8, respectively.

### Reconstructing a Combinatorial Fitness Landscape for Deoptimized Genes

**Fig. 4:**
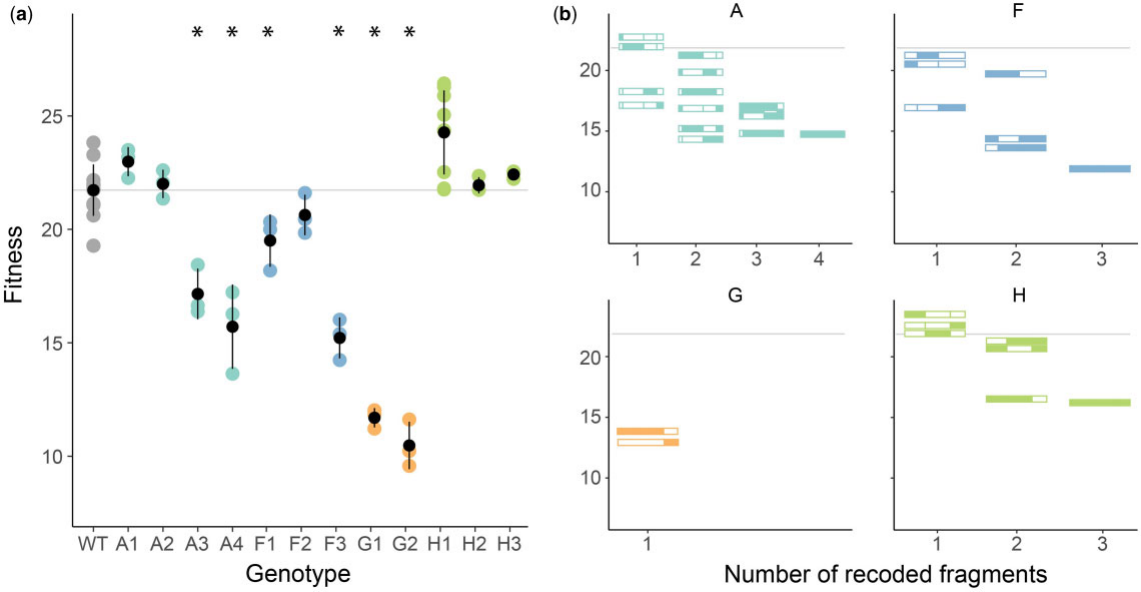
Fitness of ΦX174 when deoptimized gene fragments are combinatorially joined. The fitness of variants containing one deoptimized fragment (*a*) and all possible within-gene variants (*b*) was measured and compared with wild type (gray horizontal line). Significant differences (ANOVA, *P* < 0.01) are indicated with asterisks. In (*b*), fragment lengths are drawn to scale. Filled colors indicate the deoptimized fragments whereas unfilled blocks indicate wild-type fragments. In both (*a*) and (*b*), fitness is shown as log2-fold increase in the number of phage per hour. At least three replicates were performed for every strain (see mentary Material online). *y* axis are the same in panels (*a*) and (*b*).

We segmented the **Φ**X174 genome into 14 fragments (fig. 2) and measured the fitness of all of the possible within-gene combinations of deoptimized gene fragments (fig. 4 and see Supplementary Material online). Since genes C and J are short and encoded entirely on one fragment each, we analyzed combinations of the remaining 12 fragments. Of the 12 deoptimized strains with only one deoptimized fragment, only six have fitness values below wild type. The moderate fitness effects of these partially recoded genes allowed us to observe how deleterious effects combine. As additional deoptimized fragments are joined, the fitness of the resulting viruses decreases (fig. 4*b*). In most cases combining deoptimized fragments results in less fit viruses. The exception is gene A where instances of sign epistasis are observed. Specifically, the average fitness of A1+A3, A2+A3, A1+A3+A4, A1+A2+A4, A2+A3+A4, and A1+A2+A3+A4 are all higher than at least one of their constituent fitness values (fig. 4*b*). To further investigate how deleterious effects combine, we employed a statistical framework for calculating the best-fitting model of epistasis.

### Fitting Models of Epistasis to Combinatorial Fitness Data

The combinatorial network of genotypes that we generated in this work can be analyzed by applying simple models of epistasis (Miller et al. 2018) to determine how the effects of mutations combine. We fit the data for genes A, F, and H to three basic models—additive, multiplicative, and stickbreaking—which gave rise to no, antagonistic, and synergistic epistasis, respectively *(*see fig. 1 and Nagel et al. 2012). In fitting the three models, we conducted two analyses for each gene: one of absolute fit where we assess if the data are consistent with each model individually, and one of relative fit wherein one of the three models is assumed to be correct. The results from this analysis were not highly conclusive, but suggest the nature of epistasis is heterogeneous across different genes. For genes F and H, none of the three models could be rejected based on absolute goodness of fit (table 2). For gene F, the additive model provides the best fit to the data. For gene H, stickbreaking gives the best fit (*R*^2^ = 0.885), consistent with synergistic epistasis. This is visually clear in figure 4*b*, where the fully recoded gene H (three recoded fragments) has far lower fitness than one would expect based on the individually recoded fragments—all of which were basically neutral.

**Table 2:** Models of Epistasis Fit to Combinatorial Fitness Data

	*P* Value (absolute fit)^a^			Posterior (relative fit)^b^			*R* ^2^		
Gene	Add	Mult	Stick	Add	Mult	Stick	Add	Mult	Stick
A	0.011	0.066	<0.001	0.145	0.845	0.009	0.761	0.860	0.388
F	0.388	0.071	0.235	0.609	0.090	0.301	0.965	0.843	0.950
H	0.513	0.489	0.733	0.056	0.054	0.891	0.709	0.562	0.885

Note.—A regression of each recoded fragment’s fitness effect (against background) under each model was performed. The *P* values of each regression were combined by taking the sum of their logs. Using parametric bootstrap, the distribution of this sum was simulated. The overall *P* value is estimated by the proportion of simulations where the sum of logs is ≤ the observed value.

a Absolute goodness of fit. Small *P* values indicate that the data are inconsistent with the model (gray-filled). When a model is correct, a recoded block’s effect is uncorrelated to background fitness. The *P* value indicates how often, under parametric bootstrapping, the correlation of effect to background fitness is as strong as or stronger than that observed in the real data.

b The posterior probability assumes that one of the three models—additivity, multiplicative, stickbreaking—is correct.

**Fig. 5:**
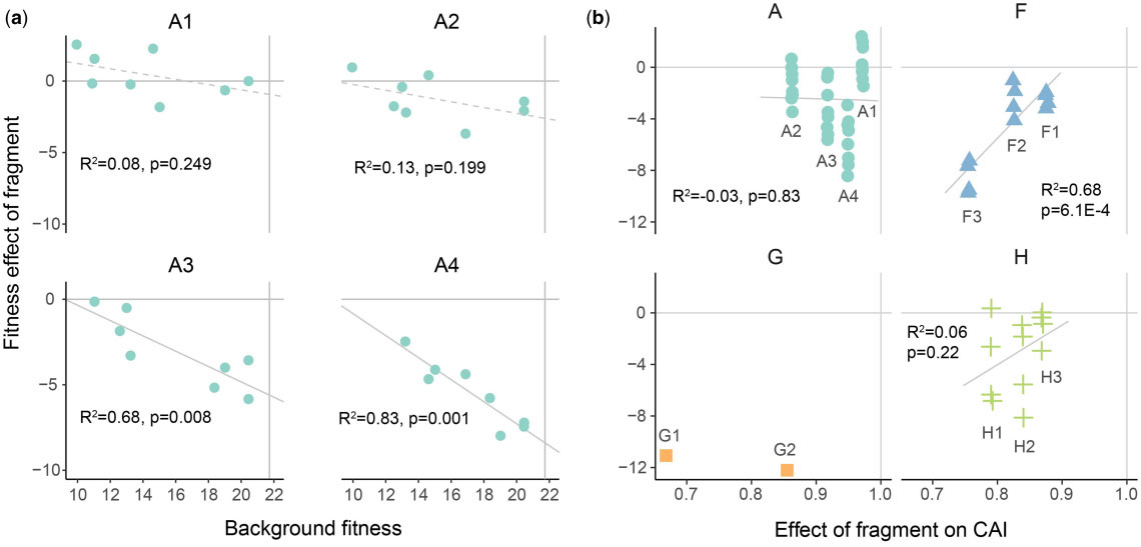
Effect of recoded fragment on all possible backgrounds. (*a*) Regressions of each recoded fragment’s fitness effect under the additive model against background fitness for gene A. Fitness effect is the difference between the background and the background plus the recoded fragment (e.g., A2 -> A1+A2, A2+A3 -> A1+A2+A3). Horizontal lines indicate a perfect fit to the additive model with no residual effects of background. Sloped regression lines indicate antagonistic/synergistic epistasis. Solid regression lines indicate that the additive model can be rejected (linear model, *P* < 0.05). The overall fit of epistatic models for each gene is shown in table 2. In (*b*), fragment fitness effects are shown against the change in CAI. Slight point jitter was used for visualization. Linear regressions are shown with *P* and *R*^2^ values. Change in CAI (Xia 2007 method) is proportional (CAI of recoded gene over CAI of background). AIC values of alternative models are shown in supplementary table S2, Supplementary Material online.

Visually, a pattern of antagonistic epistasis was observed for gene A, as several of the variants with two recoded fragments had fitness values as low as or even slightly lower than the three- and four-recoded fragment variants (fig. 4*b*). Indeed, the additive and stickbreaking models were rejected for gene A based on absolute goodness of fit (table 2). The multiplicative model, with its antagonistic pattern of epistasis, was not rejected, but the *P* value was marginal (*P* = 0.066). Strong antagonistic epistasis is occurring for gene A—even stronger than that predicted under the multiplicative model. This was revealed by regressing background fitness against fitness effect (fig. 5*a* and supplementary figs. S2–S4, Supplementary Material online). When effects were measured as differences (the additive model), negative/positive slopes corresponded to antagonistic/synergistic epistasis. Under the correct model, no correlation exists and slopes are expected to be random deviations around zero. Under the additive model (fig. 5*a*), a clear pattern across all four recoded fragments where the effect of the fragment becomes more strongly deleterious on higher fitness backgrounds (negative regression slopes) was observed. When the *P* values of the individual fragments were combined, their result is significant (supplementary fig. S2, Supplementary Material online). The analogous regression under the multiplicative model was less extreme, but even here, the slopes were consistently negative, indicating a level of antagonistic epistasis beyond multiplicative (supplementary fig. S3, Supplementary Material online).

### Correlating Codon Deoptimization to Combinatorial Fitness Data

**Fig. 6:**
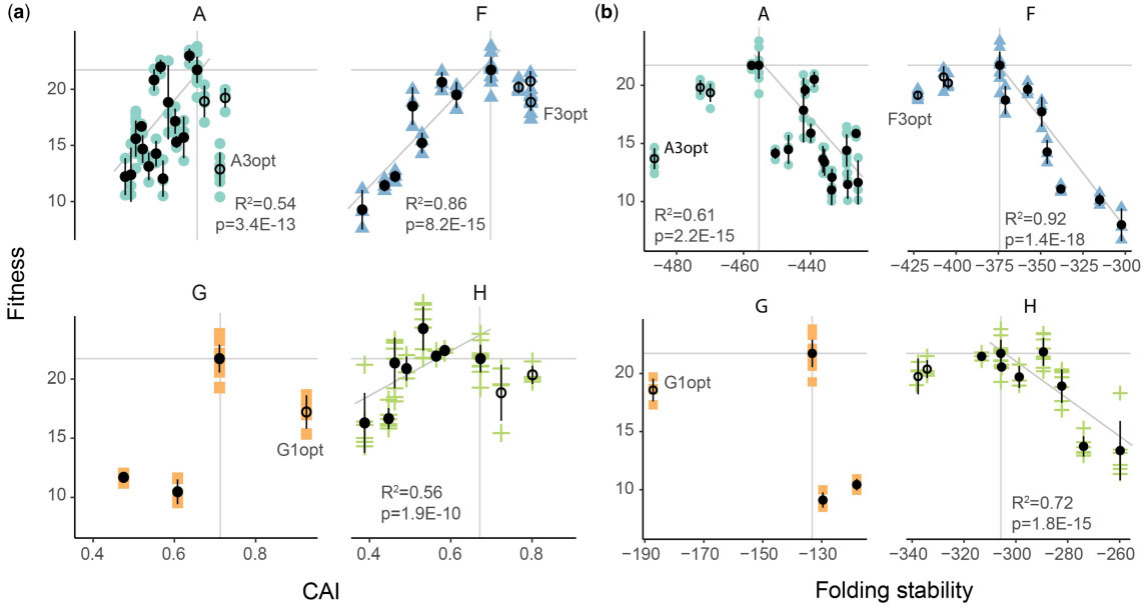
Fitness of recoded viruses correlates with codon usage bias and mRNA folding stability. (*a*) Codon usage bias (CAI) compared with fitness for viruses optimized and deoptimized in genes A, F, G, and H. Fitness and CAI of wild type are indicated with gray horizontal and vertical lines. Points to the right of these lines are optimized. Points to the left are deoptimized. (*b*) Viral fitness compared with mRNA folding stability (mfold). Wild-type values are indicated with gray horizontal and vertical lines. Less-stable transcripts (mostly deoptimized genes) have less negative values and are right of wild type. More stable transcripts (most optimized genes) have more negative values. *R*^2^ and *P* values shown are from individual (for each gene) linear regressions. A complete model comparing all indices with adjusted *P* values for multiple comparisons is shown in supplementary table S2, Supplementary Material online. Codon optimized viruses are shown with empty circles. Those significantly different from wild type are labeled (ANOVA, *P* < 0.05).

Ultimately, our goal was to correlate changes in genomic properties (e.g., codon preference) to changes in viral fitness. The most straightforward method of analysis would be to regress the two measurements, and indeed the fitness of deoptimized variants was linearly correlated to CAI even when all genes are considered together (*R*^2^ = 0.36, *P* = 2E-16, fig. 6*a*). However, it is worth noting that the data points used in this regression were not independent because the deoptimized fragments were combined to achieve higher levels of deoptimization. Our combinometric method of making variants also allowed us to correct for the cumulative fitness effect of combined fragments by calculating the effect of adding any particular fragment to different backgrounds (fig. 5*b*). For example, the effect of deoptimizing the F1 fragment was measured by comparing the fitness values of WT to deoptimized F1 (20−21 = −1), or F2 to F1+F2 (18 − 20 = −2), or F3 to F1+F3 (14 − 16 = −2), or F2+F3 to F1+F2+F3 (8 − 10 = −2). Thus, deoptimizing F1 resulted in an average fitness effect of about −2. This background subtraction approach corrected for the nonindependence of data points in regressions between change in fitness from WT and change in CAI from WT.

When we applied this correction, we observed a wide variance in fitness effects (fig. 5*b*). For example, in some backgrounds, adding deoptimized H1 reduced fitness by only _**∼**_1 doublings (dbl)/h. In other backgrounds, H1 reduced fitness by _**∼**_8 dbl/h. Despite this variation, there is a good correlation between change in CAI and change in fitness (*R*^2^ = 0.29, *P* = 0.01, fig. 5 and supplementary table S2, Supplementary Material online). Applying this background correction indicates that only a portion of the fitness changes can be explained by changes in codon usage bias. This is particularly true for genes A and H. Fragments in gene F seem to have more consistent effects (fig. 5*b*).

### How Different Synonymous Features Correlate with Fitness

We replaced **Φ**X174 codons with less-preferred codons without consideration for how alterations might affect other features in the genome. As mentioned in the introduction, many such features may be under selection. To investigate unintended consequences of codon deoptimization, we calculated numerous genome characteristics to see if any correlate with the fitness decreases observed in deoptimized fragments (see Supplementary Material online). We included many different measures of codon usage bias (CAI, tAI, *I*_TE_, etc.), codon pair bias (CPB), frequency of Shine–Dalgarno motifs, mRNA folding stability, as well as simply the number of changes made. The best predictor of fitness is the folding stability of the codon deoptimized mRNA (*R*^2^ = 0.33, p.adj = 5.1E-3), which performed better than the best measure of codon usage bias which was CAI using the Xia 2007 method (**Δ**AIC = 1.6). This correlation is easily observed when mRNA stability values are plotted against change in fitness (fig. 6*b*) and when the change in folding stability from background is regressed against the change in fitness (supplementary fig. S5, Supplementary Material online). However, many of the measures are highly correlated (see Supplementary Material online), thus we performed a stepwise regression analysis which indicated that the change in *I*_TE_, FOP, tAI, CPB, mfold, and Nc as well as the fraction of the gene edited should all be included as predictor variables in a multivariate model of fitness. This multivariate model has an adjusted *R*^2^ of 0.91 and an AIC value of 134, which is better than the best univariate predictor of fitness change (fraction of the gene edited), which has *R*^2^ and AIC values of 0.34 and 177, respectively (ANOVA, *P* = 2.1E-6). Note that CAI is not included, likely because CAI is so well correlated (*R*^2^ = 0.88) with mfold.

We were interested to see if the correlation between genomic features like CAI and fitness held up even when features were optimized, so we replaced **Φ**X174 codons with codons frequently used in *E. coli* (supplementary table S1, Supplementary Material online). In all cases, fitness was either unaffected (8/11 viruses) or reduced (3/11 viruses) (ANOVA, *P* < 0.05, fig. 6*a* and see Supplementary Material online). Because of this, if these optimized constructs are included in the regression models, the number of sites changed and fraction of gene edited become the metrics that best predict fitness from genomic measures. None of the other indices are significantly correlated with change in fitness when the optimized constructs were considered independently (linear model, *P* < 0.01), likely because most of the optimized viruses have fitness values very near wild type. We observed a peak-shaped fitness landscape when combining the optimized and deoptimized data set; this is discussed below.

## Discussion

### Patterns of Synonymous Codon Usage Biases

Synonymous codon usage biases are present in genomes across the tree of life (Grantham et al. 1980). We often think of these biases as having little consequence during the natural evolution of organisms because the strength of selection acting on a single synonymous mutation is generally weak. Nevertheless, the presence of biases shows that selection acts with sufficient strength to maintain them in the face of genetic drift. The prevailing theories on the preservation of codon biases suggest that codon choice is primarily driven by selection on translational speed and mRNA stability (Grantham et al. 1980; Robinson et al. 1984; Plotkin and Kudla 2011; Gamble et al. 2016). The enrichment of codons that use abundant tRNAs in highly expressed genes points toward a model where translation speed is correlated to tRNA abundance. We find that the most highly expressed **Φ**X174 protein ranks second best in its use of host-preferred codons, but only marginally better than the average *E. coli* gene (fig. 2). That **Φ**X174 genes are average in preferred codon usage bias according to *E. coli* usage patterns is not surprising—many viruses do not favor the most preferred host codons. Carbone (2008) also found that most **Φ**X174 genes do not use host-preferred codons and in the 116 DNA phages they studied, capsid proteins were the most codon adapted to their hosts. ssDNA phages are particularly poorly matched to their hosts’ codon biases, which is likely due to mutational pressures (Chithambaram et al. 2014). Selection could also be acting to keep viral genes from evolving to their full codon usage potential. Codon usage could control the stoichiometric expression ratio between viral genes (Cherwa et al. 2011; Quax et al. 2013), temporally regulate gene expression (Aragonès et al. 2010; Shin et al. 2015; Villanueva et al. 2016; Mioduser et al. 2017), facilitate cotranslational folding (Yu et al. 2015), dampen protein expression to avoid host immune responses (Zhao and Chen 2011; Cladel et al. 2013), be linked to global transcription patterns (Andersson and Kurland 1990; Frumkin et al. 2018), or be limited by other compositional features. Regardless of cause, codon biases among related viruses are conserved, even when they infect different hosts that have variable codon preferences (Cardinale et al. 2013; Kula et al. 2018).

### Recoding of _Φ_X174

Synonymous mutations can have substantial phenotypic effects, but it is often difficult to explain why and in what parts of a genome/gene these effects are most substantial. Our work on **Φ**X174 confirms that synonymous mutations can have massive (even lethal) fitness effects and that these effects combine in a predictable manner that is gene dependent. Although we introduced many synonymous mutations in each recoded **Φ**X174 strain, the largest observed fitness impact of any single deoptimized fragment contained only 29 synonymous codon changes. These 29 synonymous changes resulted in a 50% decrease in fitness, which is a decrease of ∼10 dbl/h or about a 1,000-fold change in the number of offspring. Kula et al. (2018) also observed phenotypic effects of deoptimizing the J gene. They observed a 25% reduction in burst size caused by 12 synonymous changes made to a 23 codon region of the J gene. Similar amounts of decrease were observed when 11 synonymous changes were made to a 22 codon region of gene F. Interestingly, over the course of 35–50 serial transfers, Kula et al. (2018) observed substitutions replacing deoptimized codons with more optimal codons in these small recoded regions. Domingo-Calap et al. (2009) also used site-directed mutagenesis to make single synonymous substitutions to **Φ**X174. None of these were lethal, but several in gene F did reduce viral fitness. Both studies suggest that codon usage is important for **Φ**X174, but neither compares effects across different regions of the genome nor do they combine multiple deoptimizing mutations. Bull et al. (2012) built increasingly deoptimized versions of the phage T7 and observed near-linear decreases in viral fitness. They deoptimized the gene encoding the T7 capsid protein, which is interesting because we also observed linear decreases for the **Φ**X174 capsid gene F. This relationship does not hold up for other **Φ**X174 genes. Jaschke et al. (2019) provides the only other combinatorial data set on par with the results presented in this article. In their study, **Φ**X174 was broken into five fragments of similar lengths. Within these fragments, synonymous changes (120 in total) were made to disrupt cryptic open reading frames and plaque size was measured for 30 combinations of the five mutated fragments. Plaque sizes were reduced about one-third, but the effects were not additive. It is difficult to directly compare this study to ours because the goal was not codon deoptimization and changes were made to multiple genes within each fragment (except fragment 1, which only contained gene A). Jaschke et al. (2019) did observe some interesting mRNA stability effects that are corroborated by our results. This is discussed in detail below.

#### Speculating on the Mechanisms Causing Reduced Fitness

In recoding **Φ**X174 with better or worse codons, we observed correlative changes in other compositional features (fig. 6 and supplementary table S2, Supplementary Material online). The best predictor of **Φ**X174 fitness was mRNA secondary structure, which is a determinant of translation rate. Tightly folded mRNA around translational start sites reduces initiation (Kudla et al. 2009; Bentele et al. 2013; Goodman et al. 2013). Folding in other parts of mRNAs slows elongation (Kelsic et al. 2016; Peeri and Tuller 2020). Translational speed is also influenced by the availability of charged tRNAs. During elongation, ribosomes must wait for cognate tRNAs. The wait-time for codons corresponding to rare tRNAs is longer than common tRNAs. The extreme result of slowed translation is ribosomal stalling and drop-off. However, faster translation is not necessarily beneficial. mRNA structure can slow translation where pausing is needed, most notably before protein structures that require cotranslational folding (Zhang et al. 2009; Yu et al. 2015; Faure et al. 2016) and at the 5′ end of a transcript, where proper loading of mRNAs onto the ribosome foreshadows correct and efficient translation (Tuller et al. 2010). Mutations that affect the folding stability and codon usage biases of bacteriophage genes tend to cause fitness effects. In the Jaschke et al. (2019) study on cryptic open reading frames in **Φ**X174, a single point mutation was identified in gene H that repeatedly evolved and ablates the detrimental effects caused by the genome-editing that they performed. An investigation of this mutation revealed that it brings the mRNA folding stability of the mutated H mRNA closer to wild type and increases H protein expression in the mutated strain of **Φ**X174. Jack et al. (2017) found that deoptimization of T7’s capsid gene (gene 10) reduced protein (but not mRNA) expression. The deoptimization of gene 10 was expected to slow translational elongation because of tRNA limitations (Jack et al. 2017, 2019). Measuring mRNA and protein expression in our deoptimized and optimized strains would provide valuable insight on the role of codon bias and mRNA stability on protein expression. Our data support the importance of mRNA folding stability for organismal fitness, as the stability of recoded **Φ**X174 genes is correlated with **Φ**X174 fitness (fig. 6*b*). Interestingly, the optimized **Φ**X174 strains almost always have increased mRNA folding stabilities and increased GC content, while folding stability and GC content are uncoupled in the deoptimized strains (folding stability decreases but GC does not). Since we did not change the 5′ end of recoded genes (confirmed by checking the folding stability of a −4 to +37 window of recoded genes), we suggest that changes to initiation rates are minimal and that the mechanisms driving decreased fitness are potentially different between optimized and deoptimized viruses. Codon deoptimization (and the resulting decrease in mRNA stability) should result in faster elongation, perhaps altering protein expression for other genes or the ratio of protein expression across the genome (see Frumkin et al. 2018 for a model of global translation). Codon optimization (and the resulting increase in mRNA stability) should result in slower elongation and decreased protein production of the targeted gene. However, the changes resulting from recoding are clearly multifaceted and require investigation aimed at understanding the mechanisms causing fitness declines. As none of the indices that we calculated fully explains the observed variance in fitness, we expect there to be other important features (e.g., cotranslational folding, ssDNA packaging, etc.) in the genome that we have not considered here.

### Combining Mutations and Epistasis

Genomes accumulate deleterious mutations over time. The detrimental effect of accumulating deleterious mutations is prevented by sex, recombination, and purifying selection which purge them from populations. In contrast to beneficial mutations which generally combine with diminishing returns (Chou et al. 2011; Couce and Tenaillon 2015), the way that the individual effects of deleterious mutations combine is less well understood. Among many issues preventing these predictions is a paucity of empirical phenotype data for networks of deleterious mutations (West et al. 1998; Kouyos et al. 2007; de Visser et al. 2011). This is especially true for combinations beyond two. A number of studies have investigated epistasis among pairs or triple sets of deleterious mutations, but the findings are mixed (Elena and Lenski 1997; Sanjuán et al. 2004; Segrè et al. 2005). Sometimes the combined effect is the sum of the individual effects (additive/no epistasis), sometimes it is less than predicted from the individual effects (antagonistic/positive epistasis) (Jasnos and Korona 2007; Guerrero et al. 2017), and sometimes it is more than predicted (synergistic/negative epistasis) (Parera et al. 2009). Among these three scenarios, antagonistic epistasis seems to be most common (Wang et al. 2002; Kouyos et al. 2007). If one considers sign epistasis to be an extreme form of antagonistic epistasis, then more support is garnished for this model as a number of studies on deleterious mutations uncover some degree of deleterious mutations becoming beneficial in combination (Lalić and Elena 2012). Our data are novel in that it builds several complete combinatorial networks of deleterious mutations, but it does lack large sample sizes. Of the networks we built, only the one for gene A had a sufficient number of data points to reject poorly fitting models. For gene A, strong antagonistic epistasis was observed. Johnson et al. (2019) recently found that this type of epistasis is common among loss-of-function mutations in yeast. They called it “increasing cost epistasis” because a given deleterious mutation tends to have a greater cost on more fit backgrounds (Johnson et al. 2019). For genes F and H, no models can be rejected, but the data suggest that mutations in gene F are additive whereas mutations in gene H combine synergistically. For the purposes of building synonymously recoded viruses for vaccines, it is promising to see gene A displaying antagonistic epistasis. With this type of epistasis where fitness flattens out, less trial and error should be required to build attenuated, but still viable, viruses.

### Synonymous Virus Genome Recoding for Vaccines

Synonymously recoding viral genomes has a potentially useful application in making live-attenuated vaccines. The antigenicity of synonymously attenuated viruses is maintained because the viral protein sequences remain unchanged. However, the process of choosing how many codons to change and what type of synonymous changes to implement is currently done without guiding principles. In fact, which synonymous features most strongly affect recoded viruses is debated (Futcher et al. 2015; Shen et al. 2015; Kunec and Osterrieder 2016). Of the dozens of viruses that have been deoptimized, a minority of them measure compositional features different from the one being directly targeted for deoptimization. At the very least, we suggest that researchers must measure a variety of compositional features when designing deoptimized constructs. A better approach would be to develop construct design software that supports researchers to engineer deoptimized viral genes (see Jorge et al. 2015 for an example using codon shuffling). This software exists for optimizing genes for expression in host cells (Grote et al. 2005; Chin et al. 2014) and may be co-opted for deoptimization purposes. In our experiments, we made no effort to isolate changes to one type of compositional feature. In exploring this possibility, we found it difficult to generate sufficient deoptimization of one feature (CAI) while keeping other features (mfold, CPB, Shine–Dalgarno frequency) unchanged. Recently, Paff et al. (2018) demonstrated that promoter ablation attenuated T7 bacteriophage in a predictable manner. Combining these edits with previous codon deoptimized strains showed increased attenuation. Targeting intragenic attenuating mutations is a promising way to test how deleterious effects combine without the added complication of trying to isolate correlative compositional features.

Like many other studies, our data showed virus codon deoptimization is an effective way to generate attenuated viruses. In cell culture and animal studies, deoptimized viruses were shown to protect from viral challenge and were stable over small numbers of passages (Martínez et al. 2016). However, much concern remains about the potential for attenuated viruses to recover virulence. It is therefore important to understand how many and what types of synonymous mutations can be made to viral genomes without completely ablating their ability to replicate in host cells. What viral genes should be attenuated? How many attenuating mutations should be made to the genome? What synonymous features should be targeted for deoptimization? In most studies to date, a limited number of deoptimized constructs (usually structural proteins) were tested. We showed that fitness decreases can be obtained by deoptimizing many of the **Φ**X174 genes, indicating that nonstructural genes may also be good targets for attenuation. One approach to avoid evolutionary reversion might be recoding multiple genes or entire viral genomes, balancing optimization and deoptimization to maintain sufficient virulence while increasing the genetic distance to wild type. This strategy could prevent recovery by mutation or by recombination with wild-type viruses. However, our work suggests that the effects of recoding will not be uniform across a genome. We found that the attenuating effects of recoding and the nature of epistatic interactions from combining fragments differ dramatically between genes.

## Materials and Methods

### Bacterial Cultures and Phage Stocks

A laboratory strain of bacteriophage **Φ**X174 (GenBank accession number AF176034) was used in this study. All experiments were carried out using *E. coli* C (strain WG5, accession number CP024090) as a host in modified Luria–Bertani media (10 g/l tryptone, 5 g/l Bacto yeast extract, 10 g/l NaCl, 2 mM CaCl_2_).

### Synthetic _Φ_X174 Genomes

*The* phage assembly platform for **Φ**X174 was used following (Faber et al. 2020). The **Φ**X174 chromosome was divided into 14 genomic fragments. Each segment is flanked by unique four nucleotide overlaps of WT **Φ**X174 sequence so that they can be amplified from the ancestral **Φ**X174 using PCR primers designed to incorporate terminal BsmB1 restriction sites. Amplicons were cloned into pCR2.1 using the Invitrogen TOPO TA cloning system (Life Technologies, Grand Island, NY). We pooled plasmid DNA containing all 14 of the phage DNA fragments in equimolar amounts and digested them with BsmB1 (Fermentas Fast Digest, Life Technologies, Grand Island, NY) for 15–30 min at 37 _**°**_C. The digested plasmids were subjected to agarose gel electrophoresis for 15 min using a 1.2% agarose gel to separate the vector from the inserts. The inserts were excised from the gel, purified using the GeneJET gel extraction kit (Fermentas), ligated overnight at 14 _**°**_C with T4 DNA ligase (Promega Corporation, Madison, WI), and transformed by electroporation into 100_** μ**_l competent *E. coli* C cells. The transformation mix was resuspended with 0.5 ml of _**Ф**_LB and plated immediately. The _**Ф**_LB was added to 3 ml of **Φ**LB top agar and plated onto a **Φ**LB agar plate. After 4–5 h of incubation at 37 _**°**_C, recombinant phage plaques were visible, and plates were removed from the incubator. Three plaques for each genotype were cored from the agar, suspended in 750_** μ**_l of **Φ**LB, and extracted off 50_** μ**_l of chloroform to kill the host cells. These stocks were used for sequencing and fitness assays. To verify that the recombinant phage contained the intended sequence, the resulting phage genome was sequenced in its entirety as previously described (Wichman et al. 2005). There was no difference in fitness between wild-type assembled phage and freezer stock phage (*P* = 0.8, *t*-test, see Supplementary Material online).

### Codon Deoptimization of _Φ_X174

Codon deoptimized and optimized fragments were synthesized in-house at the University of Texas at Austin, Applied Research Laboratories’ Gene Synthesis Facility or purchased from Biomatik USA, LLC (Wilmington, DE) according to the codon usage of five representative *E. coli* genomes (*E. coli* 536, *E. coli* UT 189, *E. coli* O157: H7 str. Sakai, *E. coli* O157: H7EDL933, and *E. coli* CFT073). Codon usage was calculated by averaging each codon’s usage frequency in CDS of these *E. coli* genomes. These hosts were chosen before the lab strain was sequenced and identified as WG5. WG5 has the same most and least-commonly used codons and would have resulted in the same recoding. Wild-type **Φ**X174 codons that could be changed to a more or less frequently used codon were exchanged for the most or least commonly used codon according to the host. Regions that we were unable to modify included overlapping genes on different reading frames, promoter regions that occurred within other reading frames, two codons at each fragment junction that contain the BsmBI sticky ends, and the region from 4,299 to 4,328 which encodes the **Φ**X174 origin of replication. In addition, six bases in front of the initiation codon (AUG) and the first 21 bases of each gene were left unmodified to assure efficient translation initiation. (See Supplementary Material online for exact coordinates of recoded regions.) Unsuccessful attempts to create live virus using synthesized fragments were repeated at least three times, then passaged in liquid culture for 24 h to allow for recovery mutants to arise.

### Measuring Viral Fitness

Fitness assays and fitness calculations were performed as previously described (Wichman et al. 2005). The assay is a determination of growth rate at low MOI in 10 ml **Φ**LB and is carried out at 37 _**°**_C. Host cells were prepared by growing to _**∼**_10^8^ cells/ml and aliquoted into 8.5 ml of warm LB just prior to adding phage. Phage from chloroform stocks were added at a concentration of 103–104 per ml. Phage fitness is expressed as the log2-fold increase in the total number of phage per hour (dbl/h). All measurements were done in triplicate. At 40 min, virus titers were determined on **Φ**LB-agar plates with 0.7% top agar. Assembled wild-type phage was used as controls.

### Calculating Genome Statistics

Calculations of genome statistics were done as follows: CAI was calculated using the seqinr cai() function in R which uses the Sharp and Li (1987) method and the *E. coli* codon usage table. An alternative form of CAI was also calculated using an updated method outlined in Xia (2007). The number of effective codons (Nc) was calculated following Wright (1990). The Index of translational elongation (*I*_TE_) was calculated according to Xia (2015). The sCAI was calculated according to Elf et al. (2003). This measure scores genes by how susceptible their codons are to a scarcity of amino-acylated tRNAs. The Xia (2007) CAI and Nc indices were calculated using Puigbò et al. (2008) with the codon table from *E. coli* WG5. tAI was calculated according to dos Reis et al. (2004) using the tAI R package (github.com/mariodosreis/tai). COUSIN18 values were calculated according to Bourret et al. (2019) using the online server at http://cousin.ird.fr/. The strength of Shine–Dalgarno sequences was included in the model by calculating the per-codon average binding strength of all Shine–Dalgarno motifs in a gene (sum of binding strengths over gene length). An empirically derived measure of translational speed was calculated according to Chevance et al. (2014) and normalized by codon family. Folding stabilities were calculated for entire gene transcripts using mfold v3.6 Mathews et al. (1999) with default parameters. Similarly, we calculated the folding stability of 42 bases (−4 to +37) around the initiation site according to Kudla et al. (2009) of each recoded gene to ensure that stability effects that might affect the initiation of translation were negligible. For figure 2, all protein-coding sequences were parsed from the *E. coli* WG5 genome (CP024090) and CAI was calculated as described above. The list of the most highly expressed genes is from Karlin et al. (2001). We compared individual (one independent variable) linear models (glm(fitness_**∼**_deltaMetric, link = “identity,” family = gaussian)) using the model.sel() function in the MuMIn R package (reported in supplementary table S2, Supplementary Material online) and global models using the stepAIC(direction = “both”) function in the MASS R package (reported in the Results section). The *P* values were adjusted using p.adjust(method = “fdr,” n = number of indices).

### Analysis of Epistasis

We analyzed the network fitness data from genes A, F, and H using the Stickbreaker R package (Miller et al. 2018) and functions therein. This package fits such data to the additive, multiplicative, and stickbreaking models. Although the additive and multiplicative models assume a mutation (a recoded block in this context) changes background fitness by a difference or a factor, respectively, the stickbreaking model assumes a mutation’s effect is scaled by the distance between the background and a fitness boundary. For fitting the stickbreaking model, we could not obtain reasonable estimates for the fitness boundary from the data (beneficial mutations are much more useful for estimating the boundary than deleterious ones). Instead, we assumed a fitness boundary of 24.5 dbl/h (wild type has fitness of 20.5); using a larger fitness boundary simply makes the stickbreaking model more like the additive model. Relative fit (posterior probabilities) was calculated following the methods in Miller et al. (2018). To estimate the absolute goodness of fit, we used parametric bootstrap. Specifically, for each gene and each model, we extracted the observed effect of each block on each background it appeared on. For each recoded fragment, we then regressed the background’s fitness against the fitness effect by fitting a simple linear model and obtained a *P* value associated with a slope of zero (illustrated for gene A in fig. 5*a*). When a model is correct, the slope of this line is expected to be zero. For a given gene and model, we take the sum of the logs of the *P* values, *P*_obs_, as a summary statistic. We noticed that the data points involved in these regressions were not independent and, as such, the *P* values were not valid. We accounted for this by simulating 10,000 data sets (using the estimated coefficient of each block and the estimated Gaussian noise parameter that captures both experimental noise and variation from model expectations). For each simulated data set, we repeated the regression for each block and combined across blocks to obtain a summary *P*_sim_. Across 10,000 simulations, this generated the approximate distribution of *P* when the model is correct. We then located *P*_obs_ in this distribution and calculated the *P* value as the proportion of simulations where *P*_sim_ < *P*_obs_ (Zhao et al. 2012; Jack et al. 2017*)*.

## Supplementary Material

evaa214_Supplementary_Data
