# Supplementary material for: ΦX174 Attenuation by Whole-Genome Codon Deoptimization
Source: Genome Biol Evol. 2020 Oct 12;13(2):evaa214. doi: 10.1093/gbe/evaa214 (PMC7881332; doi:10.1093/gbe/evaa214)
Supplement: evaa214_Supplementary_Data [file evaa214_supplementary_data.zip › codon_paper_supplementary_figs_revision_GBE.docx]

**ΦX174 attenuation by whole genome codon deoptimization**

James T. Van Leuven^1,2,‡^, Martina M. Ederer^2,‡^, Katelyn S. Slavens^1,3^, LuAnn Scott^1^, Randall A. Hughes^4,5^, Vlad Codrea^4,5^, Andrew D. Ellington^4,5^, Holly Wichman^1,2^, Craig Miller^1,2,^*

^1^Department of Biological Science, University of Idaho, Moscow, ID 83844

^2^Institute for Modeling Collaboration and Innovation, University of Idaho, Moscow, ID 83844

^3^Current address: Seattle Children's Research Institute, Seattle, WA

^4^Applied Research Laboratories, University of Texas, Austin, TX 78758

^5^Current address: Biotechnology Branch, CCDC US Army Research Laboratory, 2800 Powder Mill Rd, Adelphi, MD 20783

^6^Institute for Cellular and Molecular Biology, University of Texas, Austin, TX 78712

^‡^JTV and MME contributed equally to the work

*Corresponding author:

Craig Miller

Dept. of Biological Sciences

University of Idaho

Moscow, ID 83844-3051

crmiller@uidaho.edu

**Supplementary fig. S1 -** Heatmap showing how often particular codons were changed. The color indicates the number of edits made for codons belonging to each amino acid divided by the total number of codons changed in each fragment. Many changes are shown in pink (max=30% of changes are targeting codons for one amino acid). No changes are shown in white. The raw data is available in the supplementary materials.

**
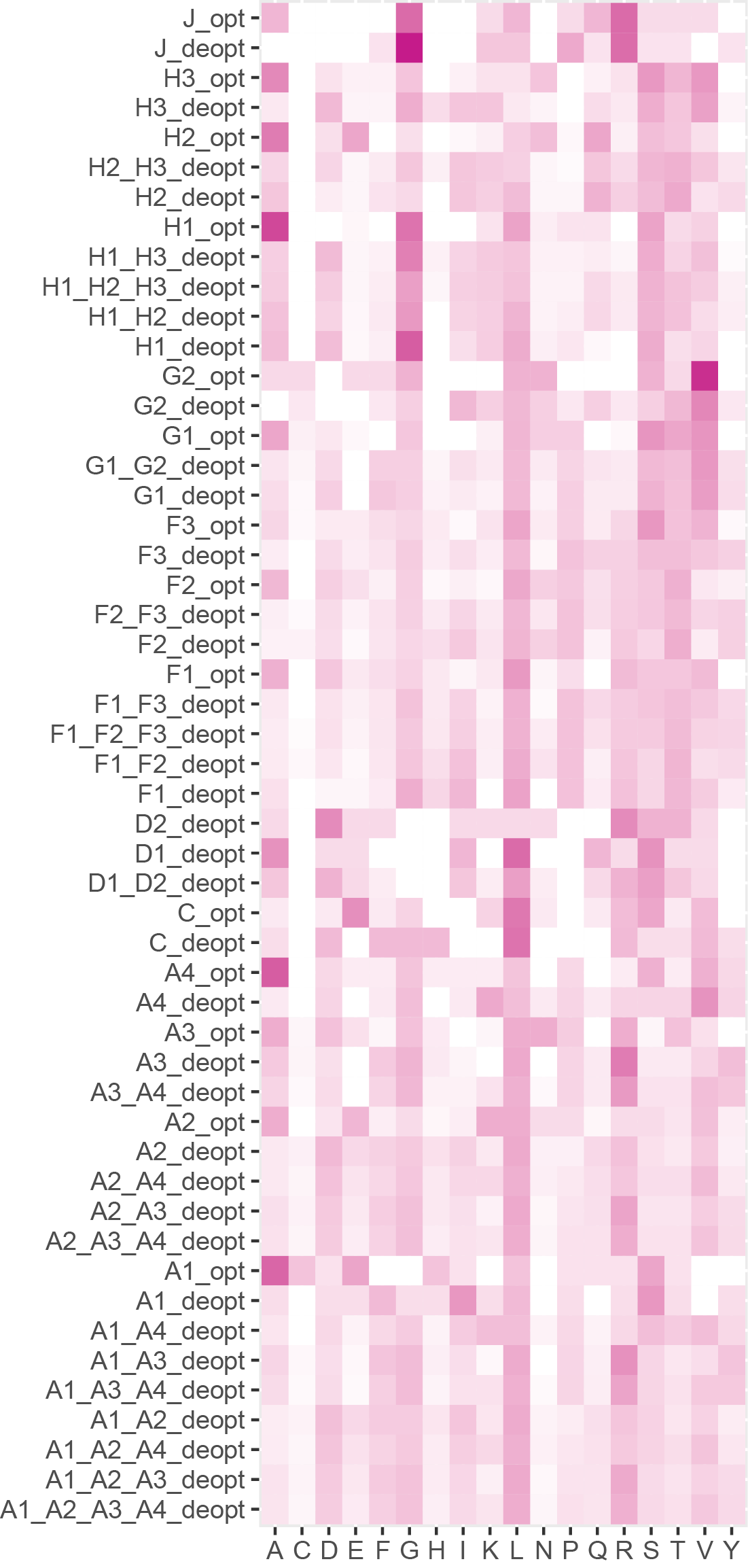
**

**Supplementary fig. S2 -** Using parametric bootstrap, the distribution of the sum of the recoded fragment’s individual p-values under the additive model (fig. 5b) was simulated. The p-value is estimated by the proportion of simulations where the sum of logs is ≤ the observed value.

**
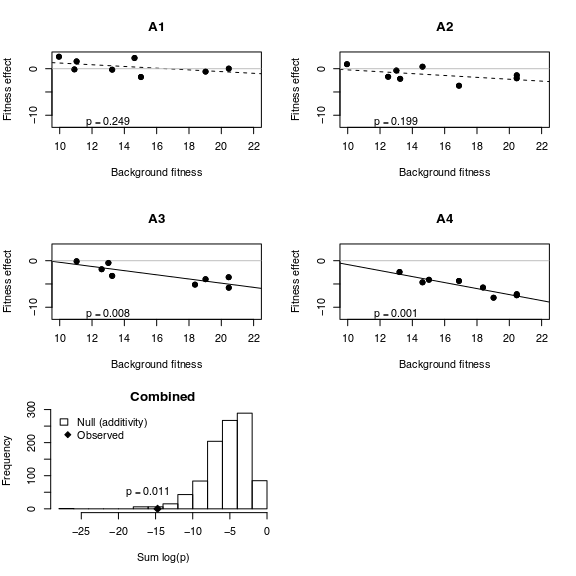
**

**Supplementary fig. S3 -** Regression of each recoded fragment’s fitness effect under the multiplicative model against background fitness. The p-values of each regression are combined by taking the sum of their logs. Using parametric bootstrap, the distribution of this sum was simulated (bottom panel). The p-value is estimated by the proportion of simulations where the sum of logs is ≤ the observed value.

**
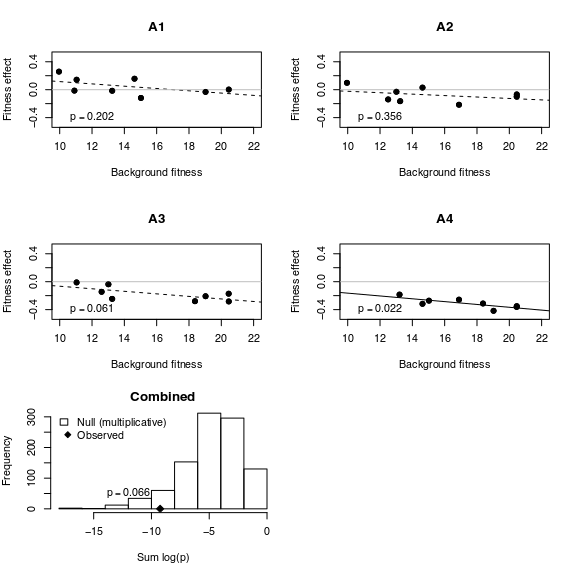
**

**Supplementary fig. S4 -** Regression of each recoded fragment’s fitness effect under the stickbreaking (synergistic) model against background fitness. The p-values of each regression are combined by taking the sum of their logs. Using parametric bootstrap, the distribution of this sum was simulated (bottom panel). The p-value is estimated by the proportion of simulations where the sum of logs is ≤ the observed value.

**
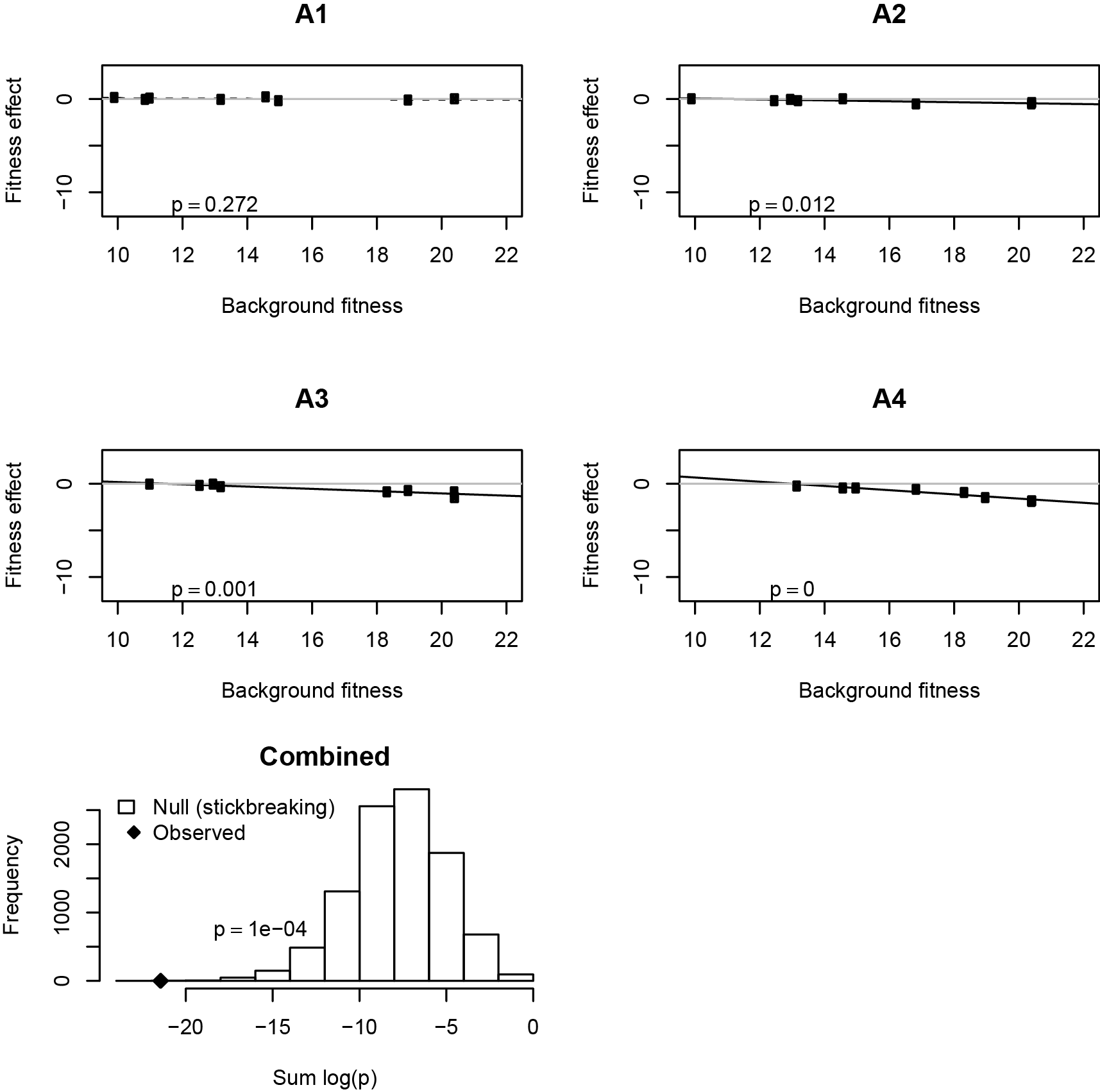
**

**Supplementary fig. S5.** Folding effect of recoding fragments on all possible backgrounds. Each fragment effects are shown against the change in folding stability. Slight point jitter was used for visualization. Linear regressions are shown with p and r-squared values. Change in folding stability is proportional. As in fig 5b, only deoptimzed genes are shown. Model AIC values are shown in supplementary table S2.

**
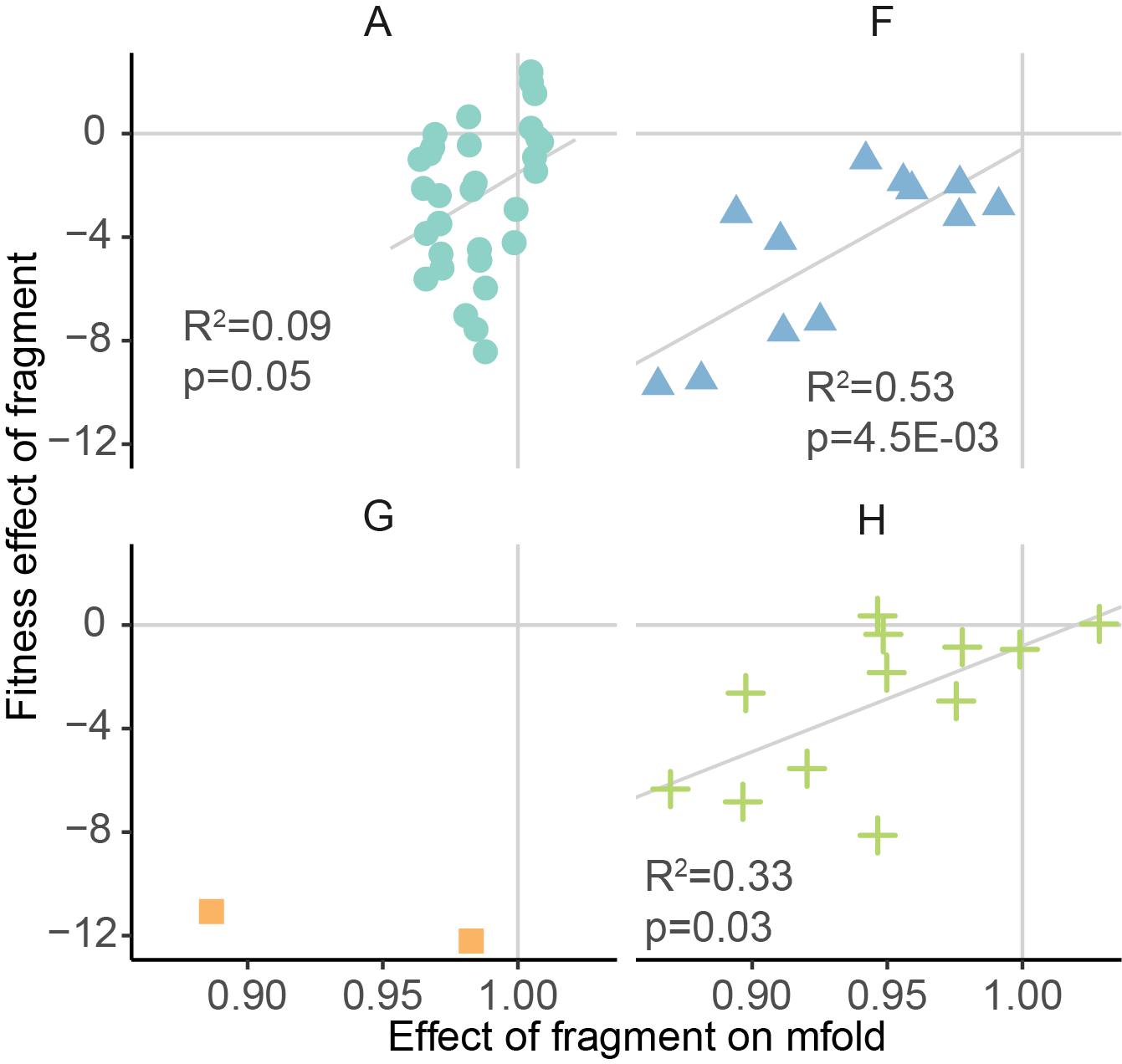
**

**Supplementary table S1 -** Edits are evenly distributed across recoded ΦX174 genes. The length of recoded genome segments, number of altered nucleotides, and number of altered codons are shown. The proportion of nucleotides edited (relative to the length of the gene or the length of the recoded fragment) are in parentheses.

|  |  | **Deoptimized** | | **Optimized** | |
| --- | --- | --- | --- | --- | --- |
| **Fragment** | **Length (bp)** | **# bp changed** | **# codons changed** | **# bp changed** | **# codons changed** |
| A1 | 90 (0.06) | 19 (0.21) | 18 | 27 (0.30) | 21 |
| A2 | 489 (0.32) | 99 (0.20) | 88 | 84 (0.17) | 75 |
| A3 | 306 (0.20) | 80 (0.26) | 64 | 69 (0.23) | 65 |
| A4 | 153 (0.10) | 37 (0.24) | 32 | 38 (0.25) | 32 |
| C | 144 (0.55) | 21 (0.15) | 20 | 40 (0.28) | 31 |
| J | 96 (0.82) | 30 (0.32) | 24 | 22 (0.23) | 20 |
| F1 | 267 (0.21) | 72 (0.27) | 65 | 68 (0.26) | 60 |
| F2 | 441 (0.34) | 115 (0.26) | 103 | 102 (0.23) | 87 |
| F3 | 552 (0.43) | 165 (0.30) | 146 | 128 (0.23) | 100 |
| G1 | 390 (0.74) | 108 (0.28) | 100 | 112 (0.29) | 86 |
| G2 | 117 (0.22) | 31 (0.27) | 29 | 21 (0.18) | 17 |
| H1 | 336 (0.34) | 89 (0.27) | 83 | 92 (0.27) | 75 |
| H2 | 393 (0.40) | 76 (0.19) | 72 | 98 (0.25) | 85 |
| H3 | 240 (0.24) | 61 (0.26) | 57 | 61 (0.26) | 47 |

**Supplementary table S2 -** General linearized models showing correlation between fitness effect and the change in various genomic features caused by recoding. See materials and methods for details on how the values were calculated. Change was calculated as the difference between the recoded gene and the wildtype gene over the wildtype gene. This normalization allowed comparisons between different genes that can have very different wildtype values. Since CPB and COUSIN have values spanning 0, we applied a constant numerical offset before calculating change. Only deoptimized constructs were used in the model. In contrast to fig. 6, all genes (including C and J) were used in this model.

| **metric** | **adj R^2^** | **pvalue** | **AIC** | **dAIC** | **padjust (FDR)** |
| --- | --- | --- | --- | --- | --- |
| Fraction of gene edited | 0.34 | 2.10E-4 | 178.8 | 0.0 | 3.57E-3 |
| Mfold change | 0.33 | 3.02E-4 | 179.5 | 0.7 | 5.14E-3 |
| RSCU change | 0.32 | 3.57E-4 | 179.8 | 1.1 | 6.06E-3 |
| I_TE_ change | 0.31 | 4.18E-4 | 180.2 | 1.4 | 7.10E-3 |
| CPB change | 0.30 | 6.17E-4 | 181.0 | 2.2 | 1.1E-2 |
| CAI (Xia2007) change | 0.29 | 6.58E-4 | 181.1 | 2.3 | 1.1E-2 |
| Nc change | 0.29 | 7.28E-4 | 181.3 | 2.5 | 1.2E-2 |
| ENC change | 0.27 | 1.20E-3 | 182.2811 | 3.5 | 2.0E-2 |
| ShineDalgarno change | 0.23 | 2.67E-3 | 183.8758 | 5.1 | 4.5e-2 |
| FOP change | 0.23 | 3.01E-3 | 184.1118 | 5.3 | 5.1E-2 |
| COUSIN_18_ change | 0.22 | 3.15E-3 | 184.2029 | 5.48 | 5.3E-2 |
| CAI (Sharp1987) change | 0.21 | 4.32E-3 | 184.8246 | 6.0 | 7.3E-2 |
| GC3 change | 0.04 | 1.51E-1 | 191.3975 | 12.6 | 1 |
| tAI change | 0.01 | 2.49E-1 | 192.1899 | 13.4 | 1 |
| sCAI change | -0.02 | 4.88E-1 | 193.1 | 14.3 | 1 |
| Empirical change | -0.03 | 6.77E-1 | 193.4 | 14.7 | 1 |
| Empirical (normalized) change | -0.03 | 8.81E-01 | 193.6 | 14.8 | 1 |

**Supplementary materials -** Spreadsheet containing (a) summary of codons targeted by recoding. The numbers show the total number of codons corresponding to any given amino acid. Another sheet shows the proportion by construct, (b) Codon preference statistics for wildtype and codon edited genes. iSD is the per codon average Shine-Dalgarno motif binding strength across a gene, (c) fitness measurements for all constructs by date and plaque number, (d) fitness measurements for freezer stock versus constructed wildtype phage, (e) genome coordinates of the recoded fragments, and (f) correlation coefficients between all of the metrics that we calculated. The wildtype fragment sequences that were synthesized and cloned into plasmid vectors are included in this table.
